# Effect of transcutaneous auricular vagus nerve stimulation on postoperative gastrointestinal dysfunction in patients with gynecological abdominal or pelvic tumors: a study protocol for a prospective, single-center, randomized, controlled clinical trial

**DOI:** 10.1080/07853890.2026.2732274

**Published:** 2026-09-18

**Authors:** Mingxia Liu, Keyu Fan, Ya Cao, Lu Qian, Yuxuan Zhang, Danru Wu, Yunfeng Wang, Chongchun Dai, Qin Zhang, Ling Zhang, Zhonghua Li, Yu Shen, Jingqiu Wei, Xi Yang, He Liu

**Affiliations:** a Department of Anesthesiology & Clinical Research Center for Anesthesia and Perioperative Medicine & Key Laboratory of Anesthesia and Analgesia Application Technology, Huzhou central Hospital, The fifth school of clinical Medicine of Zhejiang chinese Medical University, Huzhou, Zhejiang Province, China; b Huzhou Central Hospital, The Fifth School of Clinical Medicine of Zhejiang Chinese Medical University, Huzhou, Zhejiang Province, China; c Huzhou Central Hospital, The Affiliated Central Hospital Huzhou University, Huzhou, Zhejiang Province, China; d Affiliated Huzhou Hospital, Zhejiang University School of Medicine, Huzhou, Zhejiang Province, China; e Department of Education and Training, Huzhou Central Hospital, The Fifth School of Clinical Medicine of Zhejiang Chinese Medical University, Zhejiang University School of Medicine, Huzhou, Zhejiang Province, China; f Department of Anesthesiology, The Affiliated Hospital of Xuzhou Medical University, Xuzhou, Jiangsu Province, China

**Keywords:** Transcutaneous auricular vagus nerve stimulation, postoperative gastrointestinal dysfunction, gynecological abdominal or pelvic tumors

## Abstract

**Background:**

Postoperative gastrointestinal dysfunction (POGD) commonly presents with nausea, vomiting, abdominal distension, delayed flatus or stool, and prolonged intolerance to oral intake. It can cause postoperative pain, delayed gastrointestinal motility, or intestinal obstruction requiring secondary surgery, imposing substantial physical, emotional, and economic burdens. Transcutaneous auricular vagus nerve stimulation (taVNS), a non-invasive brain-gut axis modulator, may promote postoperative gastrointestinal recovery. This study will assess the efficacy and safety of taVNS in reducing the incidence of POGD after gynecological abdominal or pelvic tumor surgery.

**Methods and analysis:**

This randomized, single-center, controlled trial will enroll 210 patients scheduled for gynecological abdominal or pelvic tumor surgery and randomly assign them 1:1 to taVNS or sham-taVNS (n = 105 each). The primary outcome is the incidence of POGD within three days after surgery, assessed by the Intake, Feeling nauseated, Emesis, Exam, and Duration of symptoms (I-FEED) scoring system. Secondary outcomes include time to first flatus, time to first bowel movement, incidence and severity of postoperative nausea and vomiting, time to first ambulation, time to first oral intake, incidence of postoperative delirium, and postoperative pain intensity.

**Ethics and dissemination:**

The Ethics Committee of Huzhou Central Hospital approved this study (approval no. 202503014-03). Findings will be disseminated at national anesthesiology conferences and published in peer-reviewed journals.

**Trial registration:**

ChiCTR2500104412 (Chinese Clinical Trial Registry, http://www.chictr.org.cn; registered June 17, 2025).

## Introduction

Gynecological surgical interventions for abdominal or pelvic tumors include both radical procedures for malignant tumors and resective treatments for benign lesions. Malignant tumors, including cervical, ovarian, and endometrial cancers, together with benign conditions such as uterine leiomyomas, place a substantial burden on women’s health [1]. These procedures are often highly invasive and associated with a considerable risk of postoperative complications. The reported incidence of postoperative gastrointestinal dysfunction (POGD) after abdominal surgery ranges from 10% to 30% [2]. POGD is defined as the inability to tolerate oral intake from the end of surgery until the restoration of flatus and defecation. It is characterized by symptoms including nausea, vomiting, abdominal distension, delayed defecation, and prolonged intolerance to oral intake. The incidence of postoperative nausea may reach as high as 50%, while vomiting occurs in approximately 30%, and paralytic ileus develops in 12.9% to 32% of patients. Patients with POGD are at increased risk of complications, unplanned hospital readmissions, and mortality [2–4].

Risk factors for POGD include advanced age, cesarean section, stoma surgery, cardiac complications, and a history of abdominal surgery [2]. Surgical duration, intraoperative blood loss, fluid administration, the timing of postoperative diet initiation and mobilization, as well as perioperative opioid use, have also been shown to affect gastrointestinal recovery [3,5]. However, additional research is necessary to elucidate the inconsistent effects of these variables on the occurrence of POGD. Certain interventions, such as preoperative carbohydrate loading, postoperative coffee consumption, and gum chewing, may provide limited or inconsistent advantages in the prevention of POGD^2^. Additionally, serum bilirubin and sodium levels, combined with surgical duration, have been recognized as significant predictors of POGD in patients undergoing surgery for gynecological abdominal or pelvic tumors [4]. Therefore, the identification of these risk factors during the perioperative period is crucial, as timely interventions can contribute to reducing the incidence of gastrointestinal dysfunction, increasing patient participation in rehabilitation programs, and ultimately enhancing postoperative recovery.

Transcutaneous auricular vagus nerve stimulation (taVNS) represents an innovative and non-invasive therapeutic approach that can modulate the functions of various brainstem nuclei and higher cortical regions. The ‘brain-gut axis’ denotes the bidirectional communication network between the central nervous system and the gastrointestinal tract. This intricate system involves the vagus nerve, the sympathetic nervous system, the endocrine system, and the immune system, along with the impact of bacterial metabolites on the maintenance of gastrointestinal homeostasis. It functions to integrate the emotional and cognitive functions of the brain with peripheral intestinal regulation. Inflammatory processes exert a significant influence on the brain-gut axis, and vagus nerve stimulation has demonstrated dual anti-inflammatory effects. Specifically, taVNS activates the hypothalamic-pituitary-adrenal (HPA) axis by stimulating both afferent and efferent nerve fibers, thereby resulting in the release of glucocorticoids from the adrenal glands. Moreover, it activates the cholinergic anti-inflammatory pathway (CAP), which inhibits the release of inflammatory mediators, restores the balance between pro-inflammatory and anti-inflammatory cytokines, and contributes to the preservation of gastrointestinal function [4,6]. The primary function of the vagus nerve is to transmit sensory information from internal organs, including the liver and intestines, to the central nervous system. Moreover, it acts as a major pathway for parasympathetic signals to various organs. Activation of the parasympathetic nervous system through the vagus nerve enhances gastrointestinal motility and glandular secretion. Electrical stimulation of the auricular region has been shown to be capable of inducing gastric contractions, indicating that non-invasive vagus nerve stimulation in the auricular area can directly affect gastric activity [7]. taVNS exhibits potential in enhancing vagal activity, optimizing gastric regulation, promoting gastric peristalsis, and relieving symptoms such as constipation and abdominal pain in patients with constipation-predominant irritable bowel syndrome (IBS-C) [8,9]. Preclinical investigations suggest that taVNS notably enhances gastric emptying, improves gastric motility, reduces gastric hypersensitivity, and alleviates low-grade inflammation in animal models of functional dyspepsia (FD). Additionally, it shows therapeutic potential in modulating postoperative ileus (POI) by alleviating intestinal inflammatory responses and enhancing intestinal transit in murine models. Furthermore, taVNS has been discovered to reduce visceral hyperalgesia in mice, increase the prevalence of bifidobacterial probiotics, enhance the population of Interstitial Cells of Cajal (ICC) in the myenteric plexus region [10], accelerate upper gastrointestinal transit, and improve ICC-mediated pacing activity [11].

Currently, the scope of effective medical interventions for gastrointestinal (GI) motility disorders is limited, highlighting the need for the development of novel therapeutic strategies. taVNS, a method that regulates gastrointestinal function through the nervous system, has shown promising outcomes. However, its potential application and advantages in the context of gynecological abdominal or pelvic tumor surgeries have yet to be investigated. This study aims to evaluate the efficacy and safety of taVNS in decreasing the incidence of POGD among patients undergoing surgery for gynecological abdominal or pelvic tumors.

## Materials and methods

### Study design

The study was approved by the Ethics Committee of Huzhou Central Hospital on April 14, 2025 (approval number: 202503014-03) and was conducted in accordance with the Declaration of Helsinki. The trial was prospectively registered with the China Clinical Trial Registry on June 17, 2025 (identifier: ChiCTR2500104412). Prior to formal recruitment, a pilot study was conducted from June 18 to July 15, 2025, enrolling 40 patients (20 per group) solely to assess internal feasibility. Participants in the pilot phase were independent of the formal trial and were not included in the main analysis. Formal recruitment began on July 16, 2025. A total of 210 participants will be randomly allocated in a 1:1 ratio to either the taVNS group or the sham-taVNS group. As of June 10, 2026, the protocol version is 5.0, and the study is currently in the participant recruitment phase. All enrolled participants will undergo rigorous screening based on predefined inclusion and exclusion criteria, and written informed consent will be obtained from each participant before enrollment. The reporting of this study complies with the SPIRIT guidelines for randomized studies [12] (see Supplementary Document 1). The study timeline is presented in Table 1, and the research flowchart is illustrated in Figure 1.

**Figure 1.**
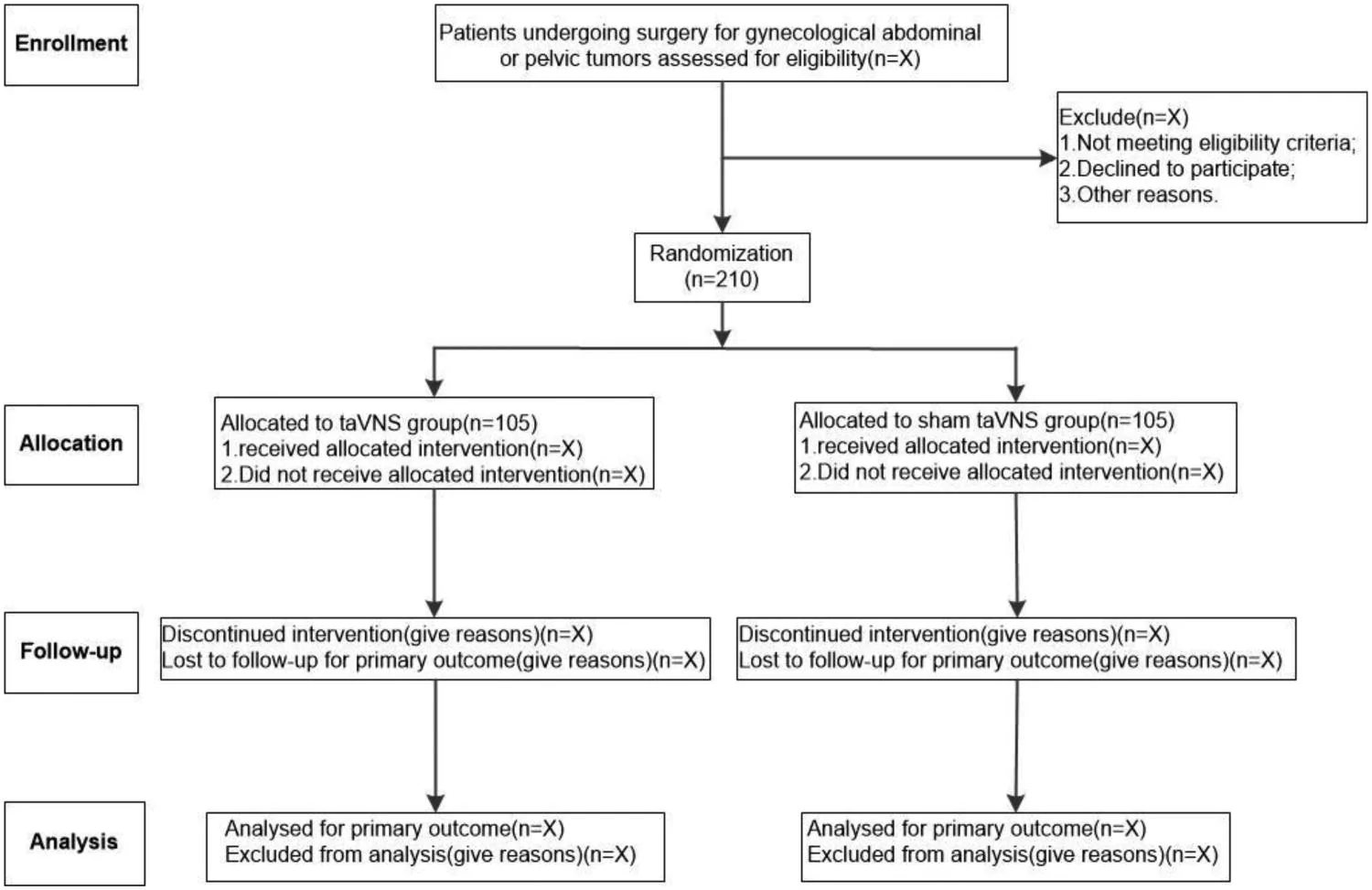
Flow diagram of the study.

**Table 1.** Schedule of patient enrollment, study interventions, and measurements complying with the SPIRIT statement.

Items	Enrollment	Allocation			Postallocation				Close-out
Time point	Preanaesthesia visit (day before surgery)	Preoperative(preparation room)	Intraoperative (following anesthesia induction)	Postoperative (anesthesia recovery room)	1 days	2 days	3 days	7days	30days
Enrollment									
Eligibility screen	X								
Preanaesthesia evaluation	X								
Informed consent	X								
Randomisation		X							
Interventions									
taVNS group		X	X	X					
sham taVNS group		X	X	X					
Assessments									
Operation time			X						
Anaesthesia time			X						
Vital signs		X	X	X					
I-FEED score	X				X	X	X		
Postoperative times for initial flatus^a^					Daily follow-up until event or POD 7				
Postoperative times for bowel movement^a^					Daily follow-up until event or POD 7				
The simplified PONV Impact Scale					X	X	X		
The initial instance of postoperative ambulation^a^					Daily follow-up until event or POD 7				
The initial instance of postoperative oral intake^a^					Daily follow-up until event or POD 7				
3D-CAM	X				X	X	X	X	
NRS score	X				X	X	X		
ERAS adherence score^b^	X	X	X	X	X	X	X		
Adverse event monitoring^c^		X	X	X	Continuous monitoring through POD 30				

SPIRIT, Standard Protocol Items: Recommendations for Interventional Trials; taVNS, Transcutaneous auricular vagus nerve stimulation; I-FEED, intake-feeling-nauseated-emesis-exam-duration of symptoms; PONV, Postoperative Nausea and Vomiting; 3D-CAM, 3-Minute Diagnostic Interview for Confusion Assessment Method; NRS, Numeric Rating Scale.

^a^Time-to-event outcomes are assessed daily (in person if hospitalized, by telephone if discharged) until the event occurs or postoperative day (POD) 7, whichever comes first.

^b^ERAS adherence score is calculated based on five protocol elements (early mobilization, early oral feeding, no routine nasogastric tube, no mechanical bowel preparation, and restrictive fluid administration).

^c^Adverse events (AEs) and serious adverse events (SAEs) are recorded from the first stimulation session through 30 days after surgery. SAEs are reported to the Ethics Committee within 24 h.

### Eligibility criteria

Researchers will identify eligible patients for the study one day prior to the scheduled surgery, in accordance with predefined inclusion and exclusion criteria. Once the eligibility of all participants is confirmed, they will be provided with a thorough explanation of the study protocol and related information.

### Inclusion criteria

1. Aged 18 years or older.
2. Scheduled to undergo elective gynecological surgery for abdominal or pelvic tumors (benign or malignant) under general anesthesia, with a confirmed preoperative pathological diagnosis or, when preoperative biopsy is not clinically indicated or feasible, a high clinical suspicion of tumor based on imaging and tumor markers.
3. American Society of Anesthesiologists (ASA) classification grades I-III.
4. Participate voluntarily and sign the informed consent form.

### Exclusion criteria

1. History of gastrointestinal motility disorders.
2. History of previous gastrointestinal surgery.
3. Diagnosis of neurological or psychiatric disorders, including a history of such conditions.
4. Skin injuries, inflammation, swelling, or infections at the intended stimulation site.
5. Individuals with visual or hearing impairments, or those with communication difficulties.
6. Severe cardiovascular or cerebrovascular diseases, or hepatic or renal dysfunction (such as severe hepatitis and renal failure).
7. The presence of pre-existing tinnitus, sinus bradycardia, or hoarseness.
8. The presence of asthma symptoms or a history of asthma.
9. Acute intestinal obstruction, intestinal perforation, or other acute medical emergencies.
10. Severe respiratory diseases including respiratory failure or severe chronic obstructive pulmonary disease.
11. Emergency surgical procedures.

### Discontinuation, withdrawal, and trial suspension criteria

To clarify participant management and data inclusion, events are categorized into four levels:

Level 1: Temporary interruption of stimulation – for mild, transient events including localized skin itching, erythema, mild chest tightness, or transient vagal reflex (heart rate 45–50 beats/min, blood pressure decrease 20–30% from baseline) that resolves with observation. Stimulation may resume upon resolution.

Level 2: Permanent discontinuation of stimulation – for severe vagal reflex (blood pressure decrease >30% from baseline, heart rate <45 beats/min with symptoms), recurrent/persistent local reactions, persistent chest/throat discomfort, or participant request. These participants remain in the ITT population and continue to be followed for outcome assessment unless they explicitly withdraw consent.

Level 3: Withdrawal from follow-up and data collection – only when the participant explicitly withdraws informed consent for all study procedures and data use, or is lost to follow-up despite reasonable contact efforts.

Level 4: Trial-level suspension – if an unexpected cluster of SAEs occurs requiring further safety investigation; decision rests with the PI in consultation with the Ethics Committee.

Consistent with the ITT principle, all randomized participants are included in the primary analysis regardless of whether they completed the intervention. Participants with Level 2 events are followed for outcomes whenever possible.

### Randomization and blinding

Randomization will be carried out using a computer-generated random number table to assign patients in a 1:1 ratio to either the taVNS group (*n* = 105) or the sham-taVNS group (*n* = 105). The taVNS group will receive active taVNS (Manufacturer: RISHENA, Model: tVNS501) throughout the perioperative period, while the sham-taVNS group will receive sham stimulation concurrently.

The randomization sequence will be generated by an independent statistician not involved in patient enrollment, intervention delivery, or outcome assessment. Allocation will be concealed using sequentially numbered, opaque, sealed envelopes prepared by the same statistician and stored in a locked cabinet in the department of clinical research. A designated research nurse, not otherwise involved in the study, will open the envelopes sequentially only after patient enrollment and baseline assessments are completed, and will be responsible for placing the stimulators, adjusting parameters, and operating the devices before, during, and after surgery according to the instructions inside the envelopes.

Data collection will be performed by four researchers blinded to group allocation across various settings, including the anesthesia operating room, surgical ward, and postoperative telephone follow-ups. Both the data collection and analysis team and the patients will remain blinded to treatment allocation. The allocation list will be kept sealed and stored in a password-protected computer file accessible only to the independent statistician until the final data analysis is completed and the database is locked. In the event of emergency unblinding, the independent statistician will be contacted to disclose the allocation, and the reason and time of unblinding will be documented.

### Sham procedure and blinding implementation

The sham-taVNS procedure is designed to mimic the active stimulation as closely as possible while eliminating the therapeutic electrical output. Both groups undergo identical electrode placement on the cymba conchae and the same intensity titration procedure to establish the maximum tolerable level. The stimulator delivers an initial 30-second pulsed stimulation with identical parameters (40 Hz, 200 μs) in both groups, after which the electrical output is automatically terminated in the sham-taVNS group [13]. This initial stimulation phase ensures that all participants experience the same transient tingling or pricking sensation, thereby supporting the credibility of the sham procedure.

To preserve blinding in practice, several measures are implemented. First, the taVNS device is identical in appearance, operation interface, and auditory feedback across both groups; the sham device has an internal circuit break that terminates effective output after the initial 30-second phase without any external indication. Second, the device is fully covered by a clean handkerchief throughout the intervention, preventing participants from observing any indicator lights or display information. Third, taVNS is delivered in a pulsed, non-sustained stimulation pattern, meaning that even in the active group, stimulation is intermittent rather than continuous. This pattern reduces the likelihood that participants can reliably infer their group assignment based on the presence or absence of sensory perception, as both groups may perceive or not perceive sensations at various time points.

To assess the effectiveness of blinding, after completion of all postoperative assessments and before data analysis, patients and outcome assessors will be asked to guess their treatment allocation (active taVNS or sham-taVNS). The proportion of correct guesses will be compared to 50% using a binomial test, and the James Blinding Index (BI) will be calculated. The BI ranges from 0 to 1, with values closer to 1 indicating more successful blinding [14]. The results will be reported alongside the primary outcome. For patients who completed the study procedures before the addition of this blinding assessment, a retrospective assessment will be conducted at the last follow-up contact before unblinding. The results will be reported separately for prospective and retrospective assessments, and a sensitivity analysis will be performed excluding retrospectively assessed patients.

### Handling of potential unblinding

In the event that a participant or outcome assessor expresses explicit knowledge of group assignment during the study, this information will be documented in the Case Report Form. Such cases will not lead to automatic exclusion; rather, they will be included in the primary intention-to-treat analysis. A sensitivity analysis will be performed excluding participants with documented unblinding to assess the robustness of the findings. Additionally, if the blinding assessment reveals systematic unblinding (e.g. correct guess rate significantly exceeding 50% in one or both groups), the primary analysis will be supplemented with a sensitivity analysis adjusting for blinding status or excluding potentially unblinded participants. The interpretation of results will consider the blinding assessment outcomes, and any limitations related to potential unblinding will be explicitly discussed.

### Emergency unblinding

During the intervention period, participants will be monitored for potential adverse events associated with taVNS, including dermatitis, local bleeding, ear pain, abdominal discomfort, and arrhythmia. An emergency unblinding procedure will be implemented to identify and manage any serious or unexpected adverse events that may be related to the intervention. The emergency unblinding will be performed by the principal investigator or a designated clinician, and any unblinding event will be documented and reported to the Ethics Committee within 24 h. This unblinding process may also be initiated in the event that a participant drops out due to suspected intervention-related adverse events or voluntary withdrawal of informed consent accompanied by safety concerns.

### Safety monitoring and adverse event reporting

Adverse events (AEs) are defined as any untoward medical occurrence in a participant enrolled in this study, irrespective of a causal relationship with the trial intervention. Serious adverse events (SAEs) are AEs that meet one or more of the following criteria: result in death; are life‑threatening; require inpatient hospitalization or prolongation of existing hospitalization; result in persistent or significant disability/incapacity; cause a congenital anomaly or birth defect. Device‑related adverse events are AEs for which a reasonable causal relationship with the taVNS device can be established, as judged by the investigator or the treating physician.

All AEs spontaneously reported by the participant or observed by the research team will be recorded from the commencement of the first stimulation session until 30 days after surgery. For each AE, the following information will be documented: description of the event, date of onset, duration, severity (mild, moderate, severe), the relationship to the device or study procedure (definitely related, probably related, possibly related, unlikely, not related), action taken regarding the study intervention, and outcome.

SAE will be reported by the principal investigator to the Ethics Committee of Huzhou Central Hospital within 24 h of becoming aware of the event. A detailed written report, including the nature of the event, its relationship to the intervention, and any subsequent management, will be submitted within seven calendar days. Device‑related SAE will additionally be reported to the local medical device regulatory authority in accordance with Chinese regulations.

The prespecified dropout criteria serve as the primary stopping rules at the individual participant level. In addition, any SAE that is judged to be causally related to the study intervention and that poses an ongoing risk to the participant will lead to permanent discontinuation of the intervention for that participant. The trial as a whole may be temporarily suspended if an unexpected cluster of SAE occurs that the principal investigator determines requires further safety investigation. The final decision regarding trial suspension or termination rests with the principal investigator in consultation with the Ethics Committee.

Because taVNS is a non‑invasive intervention with an established safety profile and this is a single‑center, low‑risk trial, an independent Data Safety Monitoring Board will not be constituted. Instead, a quarterly safety review will be conducted by the principal investigator together with a co‑investigator who is not involved in patient enrollment or outcome assessment. This review will include a summary of all AEs and SAEs, a comparison of event rates between the two groups, and an evaluation of whether the risk‑benefit balance remains favorable. Should any safety signal emerge, the principal investigator may recommend temporary suspension of recruitment pending further evaluation. At the conclusion of the study, a cumulative safety report will be submitted to the Ethics Committee.

Severe vagal reflex during taVNS is managed as follows: immediate cessation of stimulation, administration of intravenous atropine 0.5 mg, and withdrawal from the study if symptoms persist. This event is classified as an AE attributable to the device.

### Anesthesia

In accordance with the surgical protocol, eligible patients will be selected, and their medical histories will be collected. The particulars of the study procedures will also be elucidated to the patients. Informed consent for anesthesia and study procedures will be obtained (refer to Supplementary Document 2). Subsequent to the subjects signing the informed consent forms, researchers will document their comprehensive demographic data and disease-related clinical information. The sealed envelopes containing the patients’ group allocation information will be opened by operating room nurses who are not engaged in the study, and these nurses will then configure and calibrate the requisite equipment based on the group allocation information (as described below). A designated researcher will assume responsibility for gathering clinical data throughout the study.

Upon the patient’s arrival at the operating room, standard monitoring, including electrocardiogram (ECG), heart rate, pulse oxygen saturation (SpO_2_), and non-invasive blood pressure (NBP), will be applied. Subsequently, anesthesia induction and maintenance will be carried out in strict accordance with established clinical guidelines. Patients will be instructed to remain nil by mouth (8 h for solid food and 2 h for clear liquids) prior to surgery. An intravenous anesthetic regimen consisting of 1.5–2 mg/kg propofol, 0.5 μg/kg sufentanil, and 0.6 mg/kg rocuronium bromide will be administered. Endotracheal intubation will be performed 1.5 min after the administration of rocuronium bromide. Mechanical ventilation will then be initiated in pressure-controlled ventilation volume guaranteed (PCV-VG) mode, with the following initial settings: tidal volume of 6–8 mL/kg, respiratory rate of 12–15 breaths/min, an oxygen concentration of 50-80%, and an inspiratory-to-expiratory ratio of 1:2. Ventilatory parameters will be adjusted based on the target end-tidal carbon dioxide partial pressure (PETCO_2_) range of 35–45 mmHg. Anesthesia will be maintained through a combination of remifentanil (0.1–0.3 μg·kg^−1^·min^−1^), propofol (2–4 mg·kg^−1^·h^−1^), and sevoflurane (1%). Anesthetic dosages will be adjusted according to intraoperative requirements. Mean arterial pressure (MAP) will be maintained within ±20% of baseline values. Hypotension, defined as a MAP decrease of >20% from baseline, will be initially managed with intravenous fluid resuscitation. If hypotension persists, phenylephrine will be administered as an intravenous bolus of 50–100 μg (diluted to 100 μg/mL), with repeat doses every 2–5 min as needed, up to a maximum of 500 μg. Alternatively, a continuous infusion of phenylephrine (20–50 μg/min) may be initiated and titrated to effect. In cases of refractory hypotension, ephedrine 5–10 mg may be given as a rescue agent. All vasopressor administrations and corresponding hemodynamic responses will be recorded in the Case Report Form. Hypertension will be managed by adjusting the infusion rates of propofol or remifentanil; urapidil (10–25 mg) will be used for persistent elevation. Bradycardia (<40 beats/min) will be managed with intravenous atropine (0.5 mg). Intravenous fluid therapy will be individualized according to the patient’s clinical status and the judgment of the attending anesthesiologist. All patients in the taVNS and sham-taVNS groups will receive identical standardized perioperative management according to the institutional Enhanced Recovery After Surgery (ERAS) protocol for gynecological abdominal/pelvic tumor surgery [15]. Perioperative opioid use is strictly standardized *via* a uniform patient-controlled intravenous analgesia (PCIA) regimen [16]; no additional opioids are permitted outside the protocol except as rescue medication described below. Intraoperative fluid therapy follows the same goal-directed strategy for all participants. After surgery, following comprehensive assessment and confirmation of no contraindications, patients will receive flurbiprofen axetil 50 mg and palonosetron 0.25 mg. Sevoflurane, propofol, and remifentanil will then be discontinued, and extubation performed. Patients will be transferred to the post-anesthesia care unit (PACU) once they exhibit spontaneous breathing, consciousness, intact cough and swallow reflexes, ability to lift head off the pillow for ≥5 s, and a respiratory rate of 10–20 breaths/min. Transfer to the general ward occurs upon achieving a Modified Aldrete Score of ≥9. The PCIA pump is initiated at the start of skin closure, containing sufentanil 100 µg (2 mL), palonosetron 0.5 mg (10 mL), and normal saline 88 mL. Settings: loading dose 2 mL, bolus 2 mL, lockout interval 15 min, continuous infusion 2 mL/h, maximum hourly dose 10 mL. Additional non-opioid analgesics may be prescribed by the attending physician as needed.

Postoperative ward management follows the same institutional ERAS protocol. Key elements: early mobilization (out of bed on postoperative day 1), early oral feeding (clear liquids within 6–8 h after surgery, advancing to solids as tolerated), no routine nasogastric tube, no mechanical bowel preparation, and restrictive intravenous fluid administration. Gum chewing and coffee intake are not part of the standard protocol; any such practices will be recorded as co-interventions.

Rescue medications: Postoperative nausea or vomiting (PONV) is treated with intravenous metoclopramide 10 mg (repeatable once after 30 min if needed). Rescue analgesia beyond the PCIA pump is allowed, consisting of flurbiprofen axetil 50 mg or paracetamol 1 g intravenously; if inadequate, clinicians may prescribe additional opioids as needed. All rescue medication use will be documented.

The following co-interventions will be recorded in the Case Report Form: total postoperative opioid consumption (converted to intravenous morphine milligram equivalents), use of rescue antiemetics, use of laxatives, time to first mobilization, time to first oral intake, and any deviation from the ERAS protocol.

### Study interventions

A schematic diagram of the taVNS protocol is presented in Figure 2. Before the surgery, non-participating nurses open sealed envelopes that contain randomized allocation sequences to determine the parameter settings for the taVNS device according to the patient’s group assignment. Subsequently, they position the stimulation electrode in the specified auricular region of the patient. The specific operational protocols for each group are elaborated in detail below.

**Figure 2.**
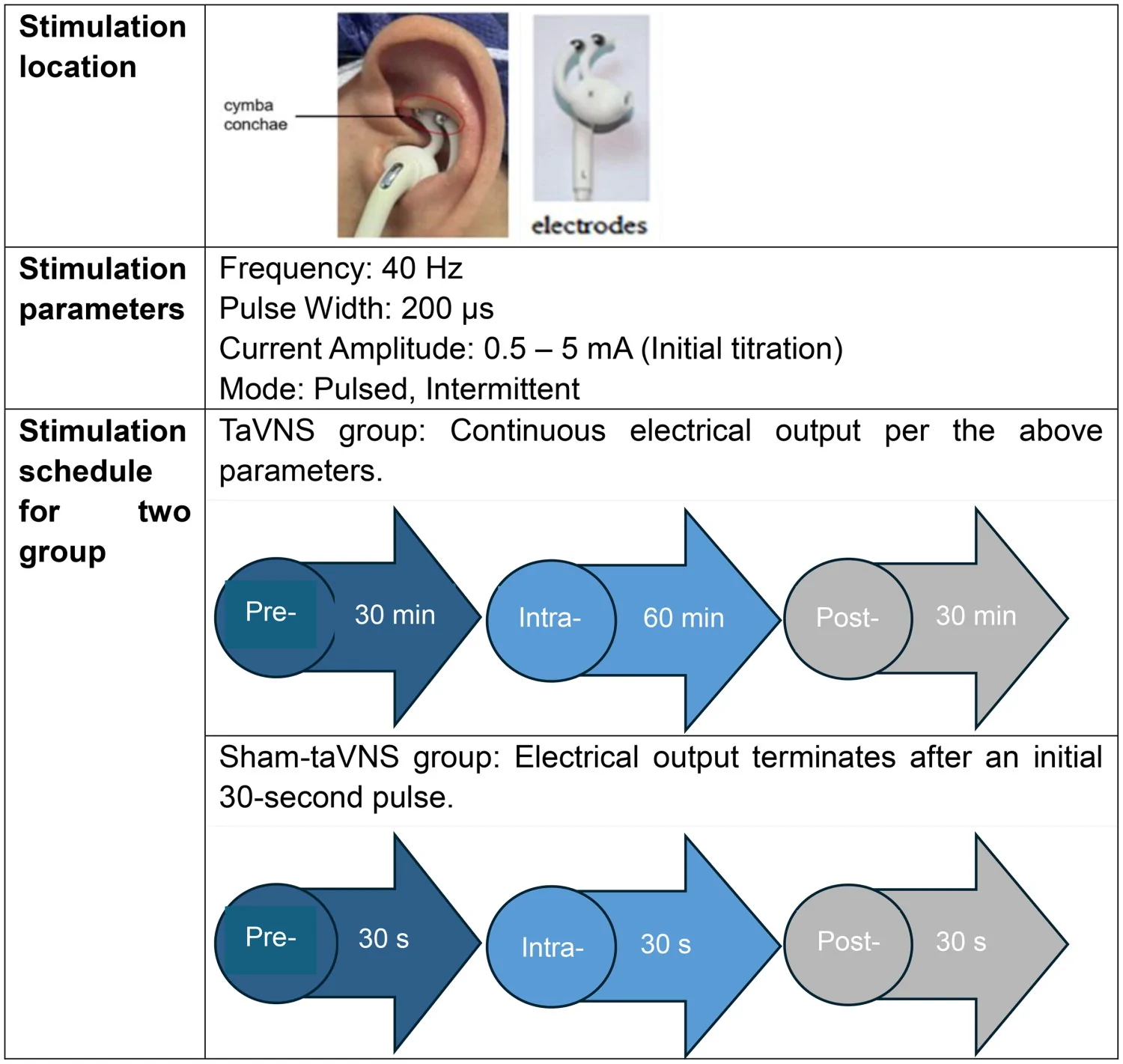
Schematic illustration of the taVNS protocol. Electrodes are placed on the left cymba conchae (indicated by the red circle). Stimulation parameters: frequency 40 Hz, pulse width 200 μs, current amplitude 0.5–5 mA (titrated to each participant’s maximum tolerable level without discomfort), delivered in a pulsed intermittent mode. The active taVNS group receives three sessions: 30 min preoperatively, 60 min intraoperatively (after anesthesia induction), and 30 min postoperatively (in the post-anesthesia care unit). The sham-taVNS group undergoes identical electrode placement and an initial 30-second stimulation, after which the electrical output is automatically terminated for the remainder of each session. Pre, preoperatively; Intra, intraoperatively; Post, postoperatively.

The taVNS group will receive taVNS at three distinct phases: preoperatively (in the preoperative preparation room), intraoperatively (after anesthesia induction), and postoperatively (in the post-anesthesia care unit). The stimulation will be specifically administered to the left ear by the research team, with the stimulation electrode positioned on the cymba conchae (Figure 2). The stimulation parameters are: current amplitude 0.5–5 mA (adjusted to the maximum tolerable level without causing ear discomfort), frequency 40 Hz, and pulse width 200 μs. The taVNS protocol comprises 30 min preoperatively, 60 min intraoperatively, and 30 min postoperatively. For the sham-taVNS group, electrode placement and initial 30-second stimulation parameters are identical to the active group, but the electrical output is automatically terminated thereafter, as described in the Sham procedure section. The device will be kept in place and fully covered by a clean handkerchief throughout the intervention.

### Outcome measures

#### Primary outcomes

The primary outcome is the incidence of POGD (maximum I‑FEED ≥6) assessed daily on postoperative days (POD) 1–3 between 8:00–10:00 AM. For patients discharged before POD3, a standardized scripted telephone interview is used. To maintain the original 3-tier I-FEED scoring, patients or caregivers are instructed to perform a self-percussion test in a supine position (one hand flat on the abdomen, tapping the back of that hand with the middle finger) to classify abdominal distension as grade 0 (no distension), 1 (visible but non-tympanic), or 3 (marked distension with tympany). To validate this telephone-based assessment, a substudy will be conducted in hospitalized patients on POD3, comparing telephone self-percussion scores against face-to-face physician examination within 2 h, using weighted Cohen’s κ (target κ ≥ 0.80). Assessment modality will be included as a covariate in sensitivity analyses [17].

#### Secondary outcomes

1. Postoperative time to first flatus and first bowel movement: This is defined as the time from the end of surgery to the patient’s first reported flatus or bowel movement, and serves as the gold standard for assessing recovery of gastrointestinal function [18]. The maximum follow-up period for these outcomes will be 7 days postoperatively. For patients discharged before day 7, events will be ascertained via daily telephone interviews until the event occurs or day 7 is reached.
2. Postoperative nausea and vomiting will be evaluated at 24,48, and 72 hours after surgery using the simplified Postoperative Nausea and Vomiting (PONV) Impact Scale. The scale comprises two components: (1) impact of nausea on recovery (0–10), and (2) vomiting episodes (0–10). A total score ≥5 defines ‘clinically important PONV’ [19].
3. Time to first postoperative ambulation: Defined as the time (hours) from the end of surgery to the first time the patient gets out of bed and walks with or without assistance [20], as documented in nursing records and patient self-reports.
4. Time to first postoperative oral intake: Defined as the time (hours) from the end of surgery to the first successful intake of clear liquids (e.g., water, broth) without significant nausea or vomiting, as documented by ward nurses.
5. Postoperative delirium: Assessed using the 3‑Minute Diagnostic Interview for the Confusion Assessment Method (3D‑CAM) at 24 hours prior to surgery, and at 24 hours, 72 hours, and 7 days after the operation [21]. The 3D‑CAM will be used exclusively for diagnosis as a binary outcome (postoperative delirium present/absent). Diagnosis requires meeting all three criteria: (1) acute onset or fluctuating course, (2) inattention, and (3) either disorganized thinking or altered level of consciousness, in accordance with the standard 3D‑CAM algorithm.
6. The Numerical Rating Scale (NRS) for postoperative pain will be evaluated at 24, 48, and 72 hours after the operation. Table 1 presents a comprehensive overview of the entire schedule.

This study aims to investigate the impact of taVNS on POGD in patients who undergo abdominal or pelvic tumor surgery. The primary objective is to evaluate whether taVNS reduces the incidence of POGD (defined as a maximum I-FEED score ≥6 within 3 days after surgery) compared with sham-taVNS (i.e. the between-group difference in the proportion of participants reaching this threshold.)

The I-FEED scoring system has been developed to provide a standardized and objective criterion for the definition of POGD [22]. It assigns scores of 0, 1, or 3 to the first four clinical parameters (intake, nausea, emesis, and abdominal examination), and 0, 1, or 2 to the fifth parameter (duration of symptoms), based on the patient’s presentation. The total scores are classified as normal (0–2), postoperative gastrointestinal intolerance (POGI, 3–5), or clinically significant POGD (≥6). In the present study, this threshold is adopted to define the primary outcome, thus facilitating a reliable comparison of dysfunction rates between the two intervention groups.

### Data collection and management

Data will be collected at three different time points—preoperatively, intraoperatively, and postoperatively—and recorded on the Case Report Form (CRF, Supplementary Document 3). A one-day preoperative anesthesia evaluation will be conducted, and informed consent will be obtained from patients during this time. The intervention will be administered by trained research personnel who are not engaged in data analysis or postoperative follow-up. These personnel will also document baseline information. Intraoperative data collection will be carried out by anesthesia researchers present in the operating room. The principal investigator will serve as the primary contact for emergencies and will provide overall supervision of the study. Personnel assigned to postoperative follow-up and data analysis will remain entirely separate from the intervention and anesthetic procedures and will be blinded to group allocation. Post-discharge follow-up will be conducted *via* telephone by trained research staff. For time-to-event outcomes, telephone follow-up will be performed daily until the event occurs or postoperative day 7, whichever comes first. All assessors underwent standardised training. Abdominal distension is graded using a unified card: 0 = flat; 1 = mild elevation without tightness; 2 = obvious distension/tympany. For symptom duration, a standardised script asks the exact date/time of first symptom, and the postoperative hour is recorded for scoring.

Preoperative data collection will be carried out one day before surgery and will include comprehensive demographic information, disease-specific characteristics, medical history, I-FEED and NRS scores, along with the documentation of any preoperative adverse events (refer to Supplementary Document 3).

Postoperative data collection will encompass a series of assessments of nausea and vomiting, Confusion Assessment Method (CAM) scores for the evaluation of delirium, and NRS scores for pain assessment. Moreover, researchers will document the time to first postoperative ambulation and resumption of oral intake. Considering the substantial influence of POGD on patients’ quality of life and recovery process, the primary outcome measure of this study is the incidence of such dysfunction. To guarantee an objective assessment, independent researchers who will not participate in anesthesia administration or intervention delivery will collect I-FEED scores once daily on the first, second, and third days after the operation. ERAS adherence will be quantified as a composite score (0–5) based on five elements: early mobilization on POD1, early oral feeding within 8 h, no routine nasogastric tube, no mechanical bowel preparation, and restrictive fluid administration. This score will be used as a continuous covariate in sensitivity analyses.

Upon the completion of the final participant’s follow-up, all data will be analyzed by independent investigators who are blinded to group allocation. Given that PONV are common manifestations of gastrointestinal dysfunction, their incidence a nd severity will be recorded separately to comprehensively evaluate the effects of taVNS on postoperative gastrointestinal function.

All investigators must complete data forms accurately and concisely. Alterations to the forms should be avoided; however, if necessary, any modifications must be signed and dated to maintain an audit trail. Data collected by stakeholders not directly engaged in the intervention shall be transferred to Microsoft Excel by the designated data management personnel.

Comprehensive documentation of all data, including detailed records of withdrawals or study discontinuations, will be maintained throughout the duration of the study. In line with local and national regulations, primary source data, including paper-based CRFs, will be securely stored by the principal investigator in a locked cabinet within the anesthesia department office, accessible only to authorized personnel. Electronic data entered into Microsoft Excel will be stored on a password-protected desktop computer in an office with restricted access, available exclusively to authorized personnel accountable for data management, processing, and analysis.

In compliance with the Chinese Good Clinical Practice (GCP) guidelines, all data related to the study must be retained for at least 10 years after completion of the study. While research findings may be published in scientific journals, patient confidentiality should be strictly safeguarded in accordance with applicable legal requirements, with all patient identifiers eliminated from published data. Personal patient information will not be disclosed unless explicitly required by relevant laws or regulatory authorities. Access to patient data may be provided to governmental regulatory agencies, institutional ethics committees, and other authorized individuals as permitted within regulatory frameworks.

As an integral part of the data collection and management for the study protocol, the final report of this study will adhere to the Consolidated Standards of Reporting Trials (CONSORT) Extension guidelines for non-drug interventions, which comply with the currently recommended standards for intervention trials [23,24].

### Sample size calculation

The sample size was calculated using PASS 15.0 software. The primary outcome for sample size determination was the incidence of postoperative gastrointestinal dysfunction (POGD) within three days after surgery, defined as an I‑FEED score ≥6.

To obtain preliminary estimates of the effect size, a pilot study was conducted from June 18, 2025, to July 15, 2025, enrolling 40 patients (20 in the taVNS group and 20 in the sham‑taVNS group). Data from this pilot study were independent of the formal trial and served solely for internal feasibility assessment; they were not pooled with the formal dataset nor included in the main analysis. The pilot results showed a POGD incidence of 20% (4/20) in the sham‑taVNS group and 5% (1/20) in the taVNS group, corresponding to an absolute risk reduction of 15%. Given the small pilot sample size, the observed effect may be inflated; therefore, a more conservative estimate was derived from published literature.

In a trial of transcutaneous auricular vagus nerve stimulation (taVNS) for laparoscopic colorectal surgery, the incidence of postoperative ileus was 20% in the control group vs. 6.25% in the taVNS group, an absolute risk reduction of 13.75% [25]. Although a recent hysterectomy study confirmed taVNS efficacy in improving I‑FEED scores in hysterectomy patients [26], that study reported median scores rather than the incidence of POGD (I‑FEED ≥6), precluding direct application of its results for sample size calculation. Therefore, we adopted the colorectal surgery estimate as a conservative reference. Assuming incidences of 20% and 6.25%, a two-sided χ^2^ test with Yates’ continuity correction (α = 0.05, power = 0.80) yielded 94 participants per group. Allowing for 10% attrition, the final required sample size was 105 per group (210 total), randomized 1:1.

### Study populations

Intention-to-treat (ITT) population: All randomized patients, regardless of compliance or completion of the study intervention.

Per-protocol (PP) population: All randomized patients who completed the study intervention without major protocol violations.

Safety population: All randomized patients who received at least one session of study intervention.

### Statistical analysis

Statistical analysis will be performed using IBM SPSS (Version 26.0), with GraphPad Prism 8.0 used for data visualization. The normality and homogeneity of variance of quantitative data will be assessed using the Kolmogorov‑Smirnov test and Levene’s test, respectively. Normally distributed data will be presented as mean ± standard deviation and compared using the independent two‑sample t‑test. Non‑normally distributed data will be expressed as median (interquartile range) and analyzed using the Mann‑Whitney U test. Count data will be presented as n (%) and compared using the chi‑square test or Fisher’s exact test as appropriate. Time‑to‑event data will be analyzed using Kaplan‑Meier curves and the log‑rank test. Repeated measurement data will be analyzed primarily using linear mixed models (LMM) or generalized linear mixed models (GLMM) as specified below. A two‑sided *p* < 0.05 will be considered statistically significant, except for secondary outcomes where a Bonferroni correction will be applied to control multiplicity.

### Missing data handling

Four types of missingness-related events are distinguished: withdrawal from intervention (retained in ITT), loss to follow-up (imputed), withdrawal of consent for data use (excluded from all analyses), and exclusion from analysis (documented).

Missing data are handled using multiple imputation (MI) under MAR with FCS (20 datasets). The binary primary outcome (I-FEED ≥6) is imputed *via* logistic regression including treatment group, baseline I-FEED, age, ASA, surgical approach, and malignancy status. Rubin’s rules are applied for pooling.

Two MNAR sensitivity analyses are pre-specified: (1) delta-adjusted MI, applying a log-odds shift (δ = 0 to 1.0, increments of 0.25) to imputed outcomes in the taVNS group; and (2) pattern-mixture model, stratifying by missingness pattern with pessimistic/neutral/optimistic assumptions. Complete-case and per-protocol analyses are also performed.

### Outcome analysis

#### Primary outcome

The primary outcome will be compared between groups using the chi-square test in the ITT population. The unadjusted odds ratio (OR) with 95% CI will be reported as the primary effect estimate. Absolute risk reduction (ARR) and number needed to treat (NNT) will also be calculated. To avoid overfitting in the multivariable analysis given the anticipated number of primary events, a parsimonious logistic regression model will be fitted including three prespecified covariates: surgical approach, malignancy status, and surgical complexity. Age and ASA will be added only if baseline imbalance exists (standardized difference >0.1). As a sensitivity analysis, Firth’s penalized logistic regression will be applied. Both unadjusted and adjusted effect estimates will be presented.

#### Secondary outcomes

Secondary outcomes are divided into confirmatory and exploratory analyses. The following are designated as confirmatory: time to first flatus, time to first bowel movement, time to first ambulation, time to first oral intake, overall PONV severity (PONV Impact Scale scores across 24–72 h), and overall pain intensity (NRS scores across 24–72 h). Exploratory analyses include time‑point‑specific comparisons of PONV and NRS at individual time points, and postoperative delirium incidence.

Time-to-event outcomes (time to first flatus, time to first bowel movement, time to first ambulation, and time to first oral intake) will be followed for a maximum of 7 days postoperatively. For gastrointestinal recovery outcomes (first flatus and first bowel movement), concurrent gastrointestinal resection will be prespecified as a competing risk, and the Fine-Gray subdistribution hazard model will be used to estimate subdistribution hazard ratios (sHR) with 95% CIs. For all time-to-event outcomes, ICU admission and reoperation are defined as intercurrent events that disrupt the natural observation period; we will perform a sensitivity analysis excluding patients who experienced these events to assess the robustness of the treatment effect estimates. Patients who discontinue the study or withdraw consent will be censored at the time of last known follow-up.The I‑FEED scores assessed at postoperative days 1, 2, and 3 will be analyzed primarily using a generalized linear mixed model (GLMM) with a cumulative logit link (ordinal logistic regression), which appropriately respects the ordinal nature of the total I‑FEED score (range 0–14). The model will include treatment group, time, and group × time interaction as fixed effects, and subject‑specific random intercepts. As a sensitivity analysis, a linear mixed model (LMM) will also be fitted, with verification of normality and homoscedasticity of residuals using Kolmogorov‑Smirnov tests, Q‑Q plots, and residual plots. Model fit will be compared between the ordinal GLMM and the LMM using the Akaike information criterion (AIC), and both sets of results will be reported. Other continuous secondary outcomes (e.g. PONV Impact Scale scores, NRS pain scores) assessed at multiple time points will be analyzed using LMM if assumptions are met, or appropriate GLMM otherwise.

Postoperative nausea and vomiting (PONV) severity scores and pain intensity scores (NRS) at individual time points (24 h, 48 h, 72 h) will be compared between groups using the Mann‑Whitney U test, as these scores are expected to be non‑normally distributed. The incidence of postoperative delirium at each assessment time point will be compared using the chi‑square test or Fisher’s exact test.

#### Multiplicity adjustment

To control the family‑wise error rate for confirmatory secondary outcomes, a Bonferroni correction will be applied. The confirmatory secondary set of outcomes consists of seven hypothesis tests: (1) group main effect for I‑FEED scores across POD 1–3; (2) group main effect for time to first flatus; (3) group main effect for time to first bowel movement; (4) group main effect for time to first ambulation; (5) group main effect for time to first oral intake; (6) group main effect for PONV Impact Scale scores across 24–72 h; and (7) group main effect for NRS pain scores across 24–72 h. The significance level for each confirmatory secondary outcome will be set at α′ = 0.05/7 ≈ 0.0071. Both unadjusted P values and Bonferroni‑adjusted P values will be reported. A confirmatory secondary outcome with an unadjusted *p* < 0.05 but adjusted *p* ≥ 0.0071 will be considered suggestive rather than conclusive. Time‑point‑specific comparisons (e.g. PONV or NRS at individual time points) and postoperative delirium analyses are designated as exploratory and are not subject to multiplicity adjustment.

#### Sensitivity analyses

To assess the robustness of the primary outcome against potential bias from different assessment modalities, we will adopt the following five sensitivity analysis strategies:

1. Analytical sets. We will compare the primary ITT analysis with: (a) a complete-case analysis restricted to participants who remained hospitalized and underwent face-to-face assessments on all three postoperative days; (b) extreme-case scenarios in which telephone-assessed abdominal distension scores are imputed as 0 (best case) and 3 (worst case) to test the direction and magnitude of potential bias; and (c) a per-protocol analysis excluding patients discharged on or before POD 2.
2. Competing risk analysis. Since early discharge (POD ≤2) may be a marker of faster recovery and could act as a competing event that prevents observation of the primary outcome within the 3-day window, we will perform a Fine-Gray subdistribution hazard model treating discharge before POD3 as a competing risk, and report the subdistribution hazard ratio (sHR) for POGD.
3. Multiple imputation under alternative missing-data assumptions.
4. Exclusion of patients with concurrent gastrointestinal resection, ICU admission, or reoperation within 3 days.
5. Firth’s penalized logistic regression for the primary outcome.

All results will be interpreted in comparison with the primary ITT analysis.

#### Subgroup analyses

Exploratory subgroup analyses will evaluate treatment effect consistency across (a) surgical approach (open vs. minimally invasive), (b) malignancy status (benign vs. malignant), and (c) surgical complexity (simple vs. complex), using subgroup-by-treatment interaction tests.

#### Exploratory mediation analysis

To explore whether the effect of taVNS on POGD is mediated by intraoperative/postoperative factors (operative time, blood loss, opioid exposure, ERAS adherence), we will conduct exploratory mediation analyses using the product-of-coefficients method with bootstrapped CIs. These are hypothesis-generating and will be interpreted as exploratory.

#### Patient and public involvement

Patients and the public were not involved in the design, conduct, reporting, or dissemination plans of this study.

## Discussion

The prevalence of benign gynecological tumors in the abdominal and pelvic cavity reaches up to 70% among women of reproductive age [27], while malignant tumors are linked to unfavorable prognoses and elevated mortality rates [28]. Surgical intervention continues to be the primary treatment approach; nevertheless, it is frequently accompanied by diverse postoperative complications. Among these, POGD - a common clinical challenge - includes postoperative ileus (POI) and postoperative nausea and vomiting (PONV). POGD is characterized by the lack of mechanical obstruction in the gastrointestinal tract and is manifested as impaired gastrointestinal motility and increased visceral sensitivity. This condition not only prolongs hospital stays and increases the likelihood of additional surgical interventions (e.g. for adhesive bowel obstruction), but also raises individual healthcare costs and burdens healthcare resources, thereby significantly hindering postoperative recovery in patients undergoing gynecological abdominal or pelvic tumor surgery.

Current clinical interventions for POGD are accompanied by significant limitations. Although nutritional support and correction of electrolyte imbalances can mitigate symptoms, they do not directly address the fundamental impairment in gastrointestinal motility or boost patients’ motivation for recovery. Invasive procedures like gastrointestinal decompression may further exacerbate patient discomfort and prolong the recovery process. While ERAS protocols advocate for early enteral feeding within 24 h post-surgery to facilitate gastrointestinal recovery [15], a portion of POGD patients encounter substantial adverse effects, including severe nausea, vomiting, and abdominal distension, which consequently restricts the viability of early feeding. Moreover, the opportunities for therapeutic intervention during anesthesia and in the immediate postoperative period (when patients are unconscious or restricted by postoperative physiological constraints such as diminished gastrointestinal motility) remain scarce. The existing gap in timely, non-invasive interventions underscores an urgent requirement for effective, well-tolerated strategies to prevent and manage POGD in the perioperative setting. taVNS has key technical and practical advantages, such as its non-invasiveness and suitability for perioperative use, which are well-adapted to this unmet clinical demand.

The primary advantage of taVNS, a non-invasive neuromodulation technique, is its capacity to be administered during anesthesia without the need for patient cooperation. This feature makes it particularly appropriate for early intervention in postoperative gynecological oncology patients. After surgery, patients frequently cannot participate in active rehabilitation measures, such as chewing gum or early mobilization, because of postoperative pain and altered levels of consciousness. Nevertheless, taVNS can be applied at three critical time points: before anesthesia induction, during the operation, and immediately after emergence from anesthesia. By initiating intervention at the onset of gastrointestinal dysfunction, taVNS may offer earlier regulatory control than conventional methods, although this advantage remains to be validated. Through the ‘brain-gut axis,’ taVNS exerts multidimensional modulation of gastrointestinal activity by transmitting afferent signals *via* the auricular branch of the vagus nerve to the nucleus tractus solitarius (NTS), which subsequently projects to the dorsal motor nucleus of the vagus (DMN) through synaptic connections [7]. This neural pathway selectively activates central regulatory centers related to gastrointestinal function. It facilitates the release of neurotransmitters like acetylcholine and motilin, promotes smooth muscle contraction, and enhances the pacemaker activity of interstitial cells of Cajal (ICC) [11], which are widely acknowledged as the pacemaker cells that govern gastrointestinal motility. Restoration of ICC function is essential for the recovery of postoperative intestinal peristalsis. Moreover, *via* the cholinergic anti-inflammatory pathway (CAP), taVNS suppresses the release of pro-inflammatory cytokines and increases the abundance of beneficial gut microbiota, including Bifidobacterium species. This mechanism raises the hypothesis that taVNS, acting through the CAP, could lower pro-inflammatory cytokines while promoting Bifidobacterium proliferation. Such reciprocal regulation might help rebalance the gut microbiota and may establish a feedback loop that directly counteracts the pathogenesis of POGD, potentially contributing to the decreased risk profile [6,10,29,30].

Several studies have investigated the role of taVNS in promoting postoperative gastrointestinal recovery [8,10]. In the colorectal surgery setting, Ru et al. reported in a randomized controlled trial of laparoscopic radical resection for colorectal cancer that a single preoperative session of taVNS significantly reduced the incidence of postoperative ileus (6.25% vs. 20%) [25], suggesting that taVNS may confer benefits even in procedures involving bowel resection. In the gynecological field, a recent randomized controlled trial by Zheng et al. in patients undergoing laparoscopic surgery for benign gynecological tumors provided preliminary evidence that taVNS significantly improved I‑FEED scores, shortened time to first flatus and defecation, and enhanced overall postoperative recovery [26]. However, that study was limited to patients with benign lesions undergoing laparoscopic procedures and did not include those with malignant tumors or open surgeries. Gynecological abdominal or pelvic tumor surgery generally does not involve gastrointestinal resection or injury, which may provide a favorable anatomical and physiological substrate for gastrointestinal recovery. To date, however, the efficacy of taVNS has not been specifically examined in a broader gynecological tumor population that encompasses both benign and malignant diseases and both laparoscopic and open approaches. Accordingly, building upon the positive findings of Zheng et al. the present protocol extends the application of taVNS to a more complex patient population, including those with malignant tumors and those undergoing open surgery. This study is designed to evaluate the effects of taVNS in this broader surgical population, although the hypothesized therapeutic benefits remain to be confirmed by the final results of this randomized controlled trial.

The I-FEED scoring system was selected as the evaluation tool for POGD in this study. It consists of five dimensions: eating tolerance, nausea, vomiting, abdominal examination results, and symptom duration. In comparison with the conventional metric of ‘time to first bowel movement,’ the I-FEED score offers a more comprehensive evaluation of patients’ clinical progression from fasting to the restoration of a normal diet. By encompassing multiple aspects of gastrointestinal recovery, the I-FEED score enables a more precise determination of patients’ preparedness to shift to regular dietary intake, thus providing enhanced guidance for clinical decision-making. In addition to the primary outcome, secondary outcomes, including postoperative delirium (assessed using the 3D-CAM) and pain (measured *via* the NRS scale), are also evaluated to explore potential additional benefits of taVNS. Both postoperative delirium [31] and pain [32] have been associated with impaired gastrointestinal function through dysregulation of the autonomic nervous system, specifically by disturbing the balance between sympathetic and parasympathetic activity. taVNS may concurrently alleviate these conditions through its regulatory effects on the central nervous system. If the findings indicate synergistic effects on pain and delirium, this would generate the hypothesis that taVNS may offer a novel approach to perioperative ‘multi-symptom integrated management,’ a possibility that warrants further investigation in dedicated trials. The limitations of this study should be objectively and systematically acknowledged. First, the selected stimulation parameters—40 Hz, 200 μs, left‑ear site, individualised intensity (0.5–5 mA), and three‑session schedule (30 min preoperative, 60 min intraoperative, 30 min postoperative)—require clearer justification with distinction of the evidence level. Human perioperative data for taVNS in gynecological tumor surgery are currently absent; therefore, the parameter selection relies on evidence from other populations and mechanistic reasoning. Evidence from human non‑surgical studies (functional dyspepsia, healthy volunteers) supports the 40 Hz frequency and 200 μs pulse width, as they enhance vagal efferent activity and improve gastric slow‑wave stability [8,33], and 30–60 min sessions are feasible and well tolerated [34]. Anatomical and safety considerations dictate the left‑ear site, which carries a lower theoretical risk of bradyarrhythmia than right‑ear stimulation in perioperative settings [35]. Animal and preclinical studies provide proof‑of‑concept that taVNS can accelerate gastric emptying, reduce inflammation, and improve intestinal transit [36], but they do not directly establish optimal parameters for human surgery. The three‑session schedule is mechanism‑based (targeting preoperative stress [37], intraoperative inflammatory surge [38], and postoperative autonomic/opioid effects^39^] rather than directly derived from existing taVNS trials, which have mostly used single‑phase stimulation [34]. We acknowledge this as a limitation and emphasize that future dose‑finding studies are needed to optimize parameters specifically for preventing postoperative gastrointestinal dysfunction in this population. Second, as a single‑center study with a moderate sample size (*n* = 210), generalizability may be limited. Nevertheless, the sample size was powered for the primary outcome based on a conservative estimate, and single‑center designs offer advantages in procedural consistency and data quality. We position this as an exploratory, hypothesis‑generating trial intended to generate preliminary evidence to inform future studies. Third, the ‘nausea level’ and ‘food tolerance’ components of the I-FEED score are based on subjective patient-reported assessments. Although this study adopts a double-blind design, using measures such as covering the stimulator and ear area with a handkerchief and ensuring that non-research personnel operated the equipment to minimize reporting bias, residual influence from subjective factors may still persist. To enhance the objectivity of outcome measurement in future studies, it is advisable to integrate objective physiological indicators, such as electrogastrography and ultrasound-based evaluations of gastric emptying time. Fourth, although a small pilot study was conducted at our center, the sample size calculation ultimately relied on effect sizes from a published trial in laparoscopic colorectal surgery rather than on the pilot data, because the limited pilot sample was considered likely to inflate the observed effect. Consequently, the effect estimates used may still be affected by population heterogeneity and publication bias, potentially overestimating the true effect and compromising the robustness of the sample size calculation. A larger randomized pilot study within the same gynecological surgical population is recommended in future research to obtain more precise and tailored parameters for sample size planning. Fifth, the sham control may be perceptible. Alternative sham strategies (e.g. low‑intensity non‑vagal stimulation) are suggested for future studies. Sixth, the strict exclusion criteria (e.g. bradycardia, asthma, severe cardiovascular disease) were designed to minimize the risk of adverse events given the novelty of taVNS in this surgical population; however, they inevitably reduce the generalizability of our findings. Once the safety profile is confirmed, future studies should consider enrolling a broader patient population with these comorbidities to better reflect real‑world clinical practice. Seventh, the inclusion of both benign and malignant tumors and varying surgical approaches (open versus minimally invasive) introduces clinical heterogeneity. Although we have prespecified subgroup analyses to evaluate the consistency of treatment effects across these strata, these analyses are underpowered. Their findings should therefore be interpreted as hypothesis‑generating, in keeping with the exploratory nature of this trial, and will require confirmation in future adequately powered studies. This study aims to evaluate whether taVNS can reduce the incidence of POGD and its related complications in patients undergoing gynecological abdominal or pelvic tumor surgery. The secondary objective is to generate hypotheses regarding potential mechanisms based on the observed clinical outcomes, which will require confirmation in future mechanistic studies. If our results demonstrate efficacy, taVNS may represent a safe, non-invasive adjunct for both preventing and managing POGD, thereby supporting wider implementation of ERAS protocols. If no significant benefit is found, systematic investigation of contributing factors will be essential to refine the role of vagus nerve stimulation in gastrointestinal motility regulation.

## Supplementary Material

supplementary document 2：Informed Consent Form.doc

Supplementary Document 3 CRF.docx

Supplementary Document 1.docx

## Data Availability

The trial protocol and registration information are openly available in the Chinese Clinical Trial Registry at https://www.chictr.org.cn (registration number: ChiCTR2500104412). After study completion, the de-identified individual participant data supporting the findings of this study will be made available from the corresponding author upon reasonable request, subject to approval by the Ethics Committee of Huzhou Central Hospital.

## Open scholarship

This article has earned the Center for Open Science badges for Preregistered. The materials are openly accessible at https://www.chictr.org.cn/showproj.aspx?proj=104412.
