# Supplementary material for: Effect of transcutaneous auricular vagus nerve stimulation on postoperative gastrointestinal dysfunction in patients with gynecological abdominal or pelvic tumors: a study protocol for a prospective, single-center, randomized, controlled clinical trial
Source: Ann Med. 2026 Sep 18;58(1):2732274. doi: 10.1080/07853890.2026.2732274 (PMC13625528; doi:10.1080/07853890.2026.2732274)
Supplement: Supplementary file 2 [file IANN_A_2732274_SM1104.docx]

**Effect of transcutaneous auricular vagus nerve stimulation on postoperative gastrointestinal dysfunction in patients with gynecological abdominal or pelvic tumors: a study protocol for a prospective, single-center, randomized, controlled clinical trial data collection table**

**version number：2.0	version date：2025.03.09**

**1、 General demographic information of patients**

number：__________	Inpatient/Outpatient Number：___________	Ward/Bed Number:__________

Name: __________	Age:_________

Height: _______ cm	Weight: __________ kg	BMI:_________

Education level: _________	Occupation: _________	Contact information:_________

Admission date: _________	Surgery date: _________	Discharge date:_________

**medical history：**

1.Surgical history: None□ Yes□ __________________;

2.Smoking history: None□ Yes□ __________________; (Years of smoking, daily smoking volume, smoking cessation status)

3.Drinking history: None□ occasionally□ often□ (years of drinking, daily alcohol consumption, whether or not quitting drinking)

4. History of PONV or motion sickness: None□ Yes□

5.Allergy：None□	Yes□	Durg□ (__________________)	Food□ (_________) Others□ (__________________)

6.Physical activity：Normal□	Restricted□	Lie in bed□

7.Diet：Normal□	excessive drinking and eating□	small amount of food intake□	unable to eat□

8.Existing diseases：__________________Hypertension：None□	Yes□; Diabetes：None□	Yes□; Coronary heart disease：None□	Yes□

9.Past medical history：__________________history of psychiatric disorders：None□	Yes□(	)

10.Family History：__________________

11.Preoperative medication (pain relief, sedation, antiemetics, etc: No□ Yes □(Drug name, dosage, and administration time)

12.Conventional drug treatment：None□	Yes□

13.Abnormal laboratory examination：None□	Yes□

14.Abnormal imaging examination：None□	Yes□

15.Preoperative I-FEED score: _____________________

Preoperative NRS score:_______________________

Preoperative adverse events: __________________

**2、 Surgical anesthesia information**

**Preoperative information: ASA grading:**_______________________

| Time  project | T1 | T2 | T3 | T4 | T5 | T6 |
| --- | --- | --- | --- | --- | --- | --- |
| Heart rate |  |  |  |  |  |  |
| MAP |  |  |  |  |  |  |

When waiting in the preoperative waiting room (T1), when the electrical stimulator stimulation starts (T2), when the electrical stimulator stimulation starts at 30 minutes (T3), when the electrical stimulator stimulation starts at 60 minutes (T4), when the electrical stimulator stimulation starts at 90 minutes (T5), and when the electrical stimulator stimulation starts at 120 minutes (T6)

Pale complexion: None□	Yes□

Sweating : None□	Yes□

Nausea, vomiting : None□	Yes□

Feeling of chest tightness : None□	Yes□

Discomfort in the throat: None□	Yes□

Individuals with localized skin itching or redness : None□	Yes□

Hypotension (systolic blood pressure below 90mmHg): None□	Yes□

Low oxygen saturation (SPO2 below 90%): None□	Yes□

Others：

**Intraoperative information:**

Surgical method: ________	Anesthesia method: ________

Extubation time: Anesthesia time: from ________ to ________, a total of _____ minutes

Surgical time: from ________ to ________, a total of _____ minutes

Will the patient be admitted to the ICU? No□	Yes□

| Time Project | T7 | T8 | T9 | T10 | T11 |
| --- | --- | --- | --- | --- | --- |
| Heart rate |  |  |  |  |  |
| MAP |  |  |  |  |  |

At the time of skin incision (T7), establishment of pneumoperitoneum (T8), 10 minutes after the start of the surgery (T9), at the end of the surgery (T10), and 5 minutes after the removal of the tracheal tube (T11)

Hypotension (systolic blood pressure below 90mmHg)：None□	Yes□

Low oxygen saturation (SPO2 below 90%)：None□	Yes□

Others：________

**Anesthetic drugs:**

| **Drugs** | **Dosage** | | | **Drugs** | | **Dosage** | | | **Drugs** | | **Dosage** |
| --- | --- | --- | --- | --- | --- | --- | --- | --- | --- | --- | --- |
| dexmedetomidine | ug | | | Fentanyl | | mg | | | rocuroniumbromide | | mg |
| Propofol | mg | | | sufentanil | | ug | | | atracuriumbesylate | | mg |
| Sevoflurane | ml | | | Remifentanil | | mg | | | cisatracurium besylate | | mg |
| pancuroniu mbromide | mg | | | Tramadol | | mg | | | Ropivacaine | | mg |
| tofisopam | mg | | | nimesulide | | mg | | | Lidocaine | | mg |
| others |  | | | others | |  | | | others | |  |
| Deoxyepinephrine | | ephedrine | | | atropine | | | others | | | |
| ug | | mg | | | mg | | |  | | | |
| Infusion volume ml | | | Blood transfusion volume ml | | | | Bleeding volume ml | | | Urine volume ml | |
| Crystal liquid：ml colloidal liquid： ml | | |  | | | |  | | |  | |

Analgesic pump: Number of incisions, length (0.2%, minimum dose of 1ml/cm):

**3、 Follow up information**

| Phase  Project | **Trial period** | | | | | |
| --- | --- | --- | --- | --- | --- | --- |
|  | Before surgery | Surgery | After surgery(day) | | | |
| Time | 1 day before surgery | On the day of surgery | 1 | 2 | 3 | … |
| I-FEED Score |  |  |  |  |  |  |
| The simplified PONV Impact Scale |  |  |  |  |  |  |
| Time of first postoperative flatus |  |  |  | | | |
| Time of first postoperative bowel movement |  |  |  | | | |
| The initial instance of postoperative ambulation |  |  |  | | | |
| The initial instance of oral intake |  |  |  | | | |
| 3D-CAM |  |  |  |  |  | |
| NRS |  |  |  |  |  | |

Remedial analgesia: None□ Yes□ _______________________ (time, medication, dosage)

Opioid related and taVNS related side effects: PONV□, itching□, dizziness□, ear irritation□, tinnitus□

**Blinding Assessment**

Patient Assessment: Which treatment do you believe you received?

□ taVNS (active stimulation)

□ Sham taVNS (sham stimulation)

□ Uncertain

Assessment nature: □ Prospective □ Retrospective

Assessor Assessment: Which treatment do you believe this patient received?

□ taVNS (active stimulation)

□ Sham taVNS (sham stimulation)

□ Uncertain

Assessment nature: □ Prospective □ Retrospective

Explicit Unblinding Record:

Did explicit unblinding occur? □ Yes □ No

If "Yes," please provide details: ________________________

Date of report: __________

Assessor’s signature: __________	Date: __________

**ERAS Adherence Score**

Early mobilization – Was the patient ambulating on postoperative day 1? (YES=1 / NO=0)

Early oral intake – Was clear liquid diet initiated within 8 hours after surgery? (YES=1 / NO=0)

No routine nasogastric tube – Was no nasogastric tube placed postoperatively? (YES=1 / NO=0)

No mechanical bowel preparation – Was mechanical bowel preparation not performed preoperatively? (YES=1 / NO=0)

Restrictive fluid management – Was a restrictive intravenous fluid protocol followed? (YES=1 / NO=0)

Total Score: ____ / 5
