# Supplementary material for: Effect of transcutaneous auricular vagus nerve stimulation on postoperative gastrointestinal dysfunction in patients with gynecological abdominal or pelvic tumors: a study protocol for a prospective, single-center, randomized, controlled clinical trial
Source: Ann Med. 2026 Sep 18;58(1):2732274. doi: 10.1080/07853890.2026.2732274 (PMC13625528; doi:10.1080/07853890.2026.2732274)
Supplement: Supplementary file 3 [file IANN_A_2732274_SM1105.doc]

**Informed Consent Form**

**version number**: 3.0	**Version Date**: 2025/4/4

**Project Name: Effect of transcutaneous auricular vagus nerve stimulation on postoperative gastrointestinal dysfunction in patients with gynecological abdominal or pelvic tumors: a study protocol for a prospective, single-center, randomized, controlled clinical trial Research institution: Huzhou Central Hospital**

**Researcher:** **department:** **Technical Title:**

Dear Sir/Madam, Hello.

We invite you (your relative) to participate in a clinical study titled " Effect of transcutaneous auricular vagus nerve stimulation on postoperative gastrointestinal dysfunction in patients with gynecological abdominal or pelvic tumors: a study protocol for a prospective, single-center, randomized, controlled clinical trial". Before deciding whether to participate in this study, please carefully read the following content, which can help you understand the study and why it is being conducted, the procedures and deadlines of the study, and the potential benefits, risks, and inconveniences that participating in the study may bring to you (your loved ones). If you have any questions or do not understand anything, please consult the researcher (doctor) until you fully understand. You can also discuss with your family, relatives, friends, etc. to help you make decisions that are in your (your loved ones') interests.

The following is an introduction to this study:

1. Research background and purpose

Gynecological surgery for abdominal and pelvic tumors, including radical surgery for malignant tumors and resection for benign tumors, is a significant health burden. The characteristics of such surgeries are often a large number of surgical invasions and complications, resulting in slow postoperative recovery, prolonged hospitalization, and increased medical costs, thereby increasing the physical, psychological, and economic burden on patients. Postoperative gastrointestinal dysfunction (POGD) has become a major concern among surgical complications, affecting numerous patients. Postoperative gastrointestinal dysfunction refers to the inability to tolerate oral intake during the postoperative period until the recovery of bowel movements and defecation. It can cause postoperative pain in patients, delay the time of oral feeding, increase the time of intravenous nutrition, prolong the time of defecation and exhaust, and even require secondary surgery for intestinal obstruction, reducing the quality of life of patients and bringing a heavy economic burden to patients, their families, and the wider society.

Transcutaneous vagus nerve stimulation is a novel non-invasive treatment technique that can regulate the activity of various brainstem nuclei and advanced brain segments. By electrically stimulating the vagus nerve in the ear, gastrointestinal motility can be regulated, gut microbiota can be improved, and visceral pain can be alleviated. Vagus nerve stimulation technology can also maintain or restore the balance between pro-inflammatory and anti-inflammatory cytokines, which helps protect the gastrointestinal tract.

At present, there is insufficient medical treatment for gastrointestinal (GI) motility disorders, and neural regulation aimed at the control of gastrointestinal function by the nervous system is a promising alternative method. This study aims to apply taVNS technology to patients undergoing gynecological abdominal and pelvic tumor surgery, and observe whether it can effectively reduce the time of first exhaust and defecation after surgery. This study will have significant implications for improving postoperative gastrointestinal dysfunction in patients.

2. Basic research content, methods, and processes

From April 2025 to January 2028, a total of 160 patients who underwent elective gynecological abdominal and pelvic tumor surgery under general anesthesia at Huzhou Central Hospital were selected. Informed consent was obtained, basic information was verified, and relevant medical history was recorded. The patients were randomly divided into two groups (experimental group and control group) according to a 1:1 ratio. TaVNS stimulation was used 30 minutes before surgery, 60 minutes during surgery, and 30 minutes after surgery. Within three days after surgery, we will conduct four postoperative follow-up visits (at 0.5 hours, 24 hours, 48 hours, and 72 hours after surgery) to collect the incidence of postoperative gastrointestinal dysfunction, the time of first exhaust, defecation, postoperative activity time, and eating after surgery, postoperative nausea and vomiting, postoperative delirium, postoperative pain. the need for additional pain relief afterwards.

3. What do you (your family) need to do if you participate in the research

You need to fully understand the details of this study and voluntarily decide whether to participate. If you agree to participate in this study, you need to sign this informed consent form and agree to our use of your medical information. Cooperate with us for relevant operations and postoperative follow-up.

4. The benefits that participating in this study may bring to you (your loved ones) and society

You may reduce the reduce the incidence of postoperative gastrointestinal dysfunction and accelerate the recovery of postoperative gastrointestinal function by participating in this study. Of course, you may not be able to benefit from this study. However, based on your data during the research period, it can help researchers further understand the role of the vagus nerve in postoperative gastrointestinal function, choose a more reasonable perioperative plan to prevent and treat postoperative gastrointestinal dysfunction, and benefit your future diagnosis and treatment as well as the same patients.

5. Possible adverse reactions, discomfort, and risks associated with participating in this study, as well as prevention and treatment measures

During the intervention period, dermatitis may occur. The stimulation was stopped and observation was conducted. If there was no improvement, a consultation with a dermatologist was sought for medication treatment; If there is local bleeding in the stimulated area, stop the stimulation, press to stop bleeding, and disinfect; If there is pain in the ear, stop stimulating and observe. If there is no improvement, please consult with an otolaryngologist for treatment; During the use of the taVNS device, electrocardiogram monitoring will be performed，and observe the stimulation of the surrounding skin; When damage related to research occurs, we will actively seek treatment and consult with relevant experts. The expenses incurred will be borne by the research team.

6. Costs and compensation

(1). Operations and inspections related to research are free of charge;

(2). It is recommended to compensate for damages related to research in accordance with relevant Chinese laws and regulations.

7. Alternative solutions

Not participating in this study will be treated according to normal diagnosis and treatment.

8. Your authority

Whether or not to participate in this study depends entirely on your voluntary participation. You may refuse to participate in this study or withdraw from the study without reason at any time during the study process, which will not affect your (your family's) relationship with the doctor, nor will it affect the loss of your (your family's) medical or other benefits. You also have the right to be informed, confidential, compensated, receive free treatment and compensation in case of damage, obtain new information, re sign new versions of informed consent forms, and obtain informed consent forms. If you have any questions about this study or have any problems during the research process, please contact the researcher xxx of this project at xxx.

9. Regarding personal information and research data

Your (your relative's) medical records (including research medical records and physical and chemical examination reports, etc.) will be kept in the hospital according to regulations. Except for researchers, ethics committees, monitoring, inspection, and drug administration departments, other personnel unrelated to the research have no right to access without permission. The public report of the results of this study will not disclose your (your relative's) personal information, and the research results will not be fed back to you (your relatives). We will make every effort to protect your (your family's) personal information and related data from being leaked (including secondary use) in accordance with the law.

10. Regarding biological samples

This study does not involve biological samples.

11. Terminate participation in the study

Due to the following reasons, your (your relative's) participation in this study may be terminated:

(1). You do not follow the doctor's advice.

(2). You have experienced a serious condition that may require treatment.

(3). The research doctor believes that terminating the study is most beneficial for your health.

(4). You withdraw your consent.

12. Ethics Committee

This study has been reported to the Medical Ethics Committee of Huzhou Central Hospital and has been reviewed and approved by the committee. During the research process, matters related to ethics and rights can be addressed by contacting the Medical Ethics Committee of Huzhou Central Hospital.

Phone: 0572-2709719 Email Address: hzszxyyll@163.com

**Researcher statement:**

I confirm that I have explained the details of this study, including their rights and potential benefits and risks, to the research participants (and/or their guardians/authorized agents); impartial witnesses (if applicable), answered their questions, and the research participants (and/or their guardians/authorized agents); impartial witnesses (if applicable) have expressed their understanding of my explanation and clarification. I have provided a signed copy of the informed consent form to the study participants (or guardians).

Signature of researcher: Interview time: YYYY-MM-DD at ________

Contact phone number:

**Research participants (or guardians/authorized agents/impartial witnesses) declare:**

I confirm that I have read the informed consent form for this study, and the researcher has provided me with a detailed explanation of the relevant content, answered my questions, and helped me understand the terms and conditions. I also confirm that if I (my family member) do not participate in this study or withdraw at any time during the study, it will not have a negative impact on my (my family member's) medical activities and doctor-patient relationship, or cause any other harm to my (my family member's) interests. I understand that the public reporting of the results of this study will not disclose my (my family's) personal information. I have ample time to consider. After careful consideration, I have decided to participate in this study and agree to publicly report the results of this study using my (my relative's) relevant diagnosis and treatment data and information.

Signature of research participant: Signature time: YYYY-MM-DD at ________

Contact phone number:
